# Radiative forcing by light-absorbing aerosols of pyrogenetic iron oxides

**DOI:** 10.1038/s41598-018-25756-3

**Published:** 2018-05-09

**Authors:** Akinori Ito, Guangxing Lin, Joyce E. Penner

**Affiliations:** 1 Yokohama Institute for Earth Sciences, JAMSTEC, Yokohama, Kanagawa 236-0001 Japan; 2 Pacific Northwest National Laboratory, Richland, WA USA; 3 Department of Climate and Space Sciences and Engineering, University of Michigan, Ann Arbor, Michigan USA

## Abstract

Iron (Fe) oxides in aerosols are known to absorb sun light and heat the atmosphere. However, the radiative forcing (RF) of light-absorbing aerosols of pyrogenetic Fe oxides is ignored in climate models. For the first time, we use a global chemical transport model and a radiative transfer model to estimate the RF by light-absorbing aerosols of pyrogenetic Fe oxides. The model results suggest that strongly absorbing Fe oxides (magnetite) contribute a RF that is about 10% of the RF due to black carbon (BC) over East Asia. The seasonal average of the RF due to dark Fe-rich mineral particles over East Asia (0.4–1.0 W m^−2^) is comparable to that over major biomass burning regions. This additional warming effect is amplified over polluted regions where the iron and steel industries have been recently developed. These findings may have important implications for the projection of the climate change, due to the rapid growth in energy consumption of the heavy industry in newly developing countries.

## Introduction

Iron (Fe) oxides in mineral dust have been shown to strongly absorb solar radiation^1–3^. In desert soils, Fe oxides are generally hematite (α-Fe_2_O_3_) and goethite (FeOOH), which cause soil-derived dust absorption at ultraviolet (UV) and visible wavelengths^4,5^. These two minerals have distinct optical properties that cause different intensities of shortwave absorption and thus radiative forcing (RF) of aerosols^6,7^. Further, the physical and chemical properties of Fe oxides in minerals are different between aeolian dust and fly ash^8–11^. Moreover, the distinct emission sources of pyrogenic Fe oxides have been shown to enhance Fe bioavailability due to atmospheric processes and cause potentially harmful effects on human and ocean health^12–15^.

Fe speciation in fly ash is dependent on the emission source^16,17^ and is also influenced by atmospheric processing^13,14,18,19^. Ferric sulfate and aggregated nanocrystals of magnetite (Fe_3_O_4_) are dominant components of Fe in oil fly ash^16,17^. In coal fly ash, Fe oxides are distributed between crystalline and aluminosilicate-glass phases during ash formation^8–11^. The enrichment of Fe aggregates attached to the surface of particles has been observed for Fe-rich fly ashes^9,10^. In contrast to Fe oxides at emissions from combustion sources, the Fe in aged fly ashes is coated in the form of Fe sulfate due to strong acidity of sulfate in fine particles^18,19^. Laboratory experiments suggested that labile Fe in coal fly ash was mainly transformed from the aluminosilicate-glass phase^12,13^. An atmospheric chemical transport model that implemented Fe chemistry for combustion aerosols suggested a significant transformation of Fe from insoluble to labile form during atmospheric photochemical processing^14^. After the dissolution of Fe from aerosols, nanoparticles can form via the precipitation of ferrihydrite from colloidal solutions under higher pH conditions in simulated cloud processing^20^.

It has been suggested that Fe oxides in the form of aggregated magnetite nanoparticles from anthropogenic sources contributed between 4 and 7% of the shortwave absorption (mW m^−3^) of black carbon (BC), based on aircraft measurements over the Yellow Sea and East China Sea^21^. The refractive index of black-colored magnetite is more similar to BC than is hematite and goethite. Thus dark Fe-rich mineral particles that originate from combustion sources can be more effective at absorbing sun-light than is that from soils in arid and semi-arid regions. The significant correlation between the number concentrations of Fe oxides and BC suggests that the spatial distribution of the emission flux of Fe oxides over the East Asian continent is similar to that of BC^21^. However, the contribution of different sources of Fe to total Fe in combustion aerosols remains uncertain. Magnetite is crystallized from molten silicates and is partly trapped in the aluminosilicate-glass phase during ash formation, while the magnetic fraction of fly ash can be collected by electrostatic precipitator if the emission control device is equipped and maintained properly^8–10^. Efficient filtering technologies may prevent environmental magnetic pollution of airborne particulate matter (PM)^15^ but efficient filtering is not expected in newly industrializing countries with fast growing economies.

In wildfires, it is well known that Fe oxides in soils are transformed to magnetic Fe oxides at high temperature^22,23^. Spherical particles originating from a biomass-burning event showed an enrichment of the reduced form of Fe(II) in the outer shell of the particles^24^. Moreover, the effects of burning on soil color are evident^25^. Alteration of Fe(III) oxides (goethite and hematite) to Fe(II, III) oxides (magnetite) due to fires has been documented in mineral soils containing organic matter^26,27^. Laboratory experiments suggest a two-step reaction, that is, the transformation of goethite to hematite via dehydoxylation and the subsequent reduction to magnetite in the presence of organic matter that acts as a reductant^27^.

Previous modeling studies have emphasized the role of soil-derived Fe oxides in RF^1–3^ and that of combustion sources in bioavailable Fe deposition^28–30^. However, there is no estimate of RF by pyrogenic Fe oxides, despite its potential importance^21^. Here, we hypothesize that Fe-rich aerosols from pyrogenic emissions are important sources of light-absorbing aerosols. To test this hypothesis, we use a global chemical transport model and a radiative transfer model to estimate the RF of Fe oxides from combustion sources compared to that of BC. The description of the models is provided in the Methods.

## Results and Discussion

**Figure 1:**
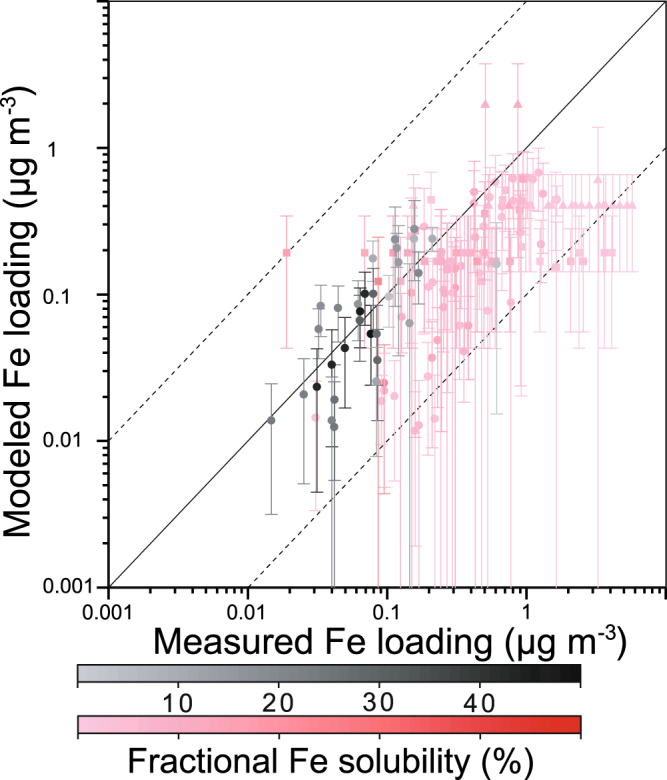
Comparison of simulated and observed values for atmospheric loading of aerosol Fe (μg m^−3^) in PM_2.5_ (black colors) and total PM (red colors) at the GOSAN site (33°N, 126°E)^33^ (triangles), East China Sea^34^ (squares), and the Bay of Bengal^35^ (circles) near the surface (averaged lowest three model layers). The black solid line represents a 1-to-1 correspondence. The black dashed lines show deviations from the solid line by a factor of ±10. The vertical lines in symbols correspond to ±1 standard deviation based on the daily modeled averages in 2014. The color gradient in symbol denotes the fractional Fe solubility of measurements. This map was created in Origin Pro 2017.

We compared BC concentrations near the surface (averaged lowest three model layers) with measurements in China, Korea, and Japan in 2014^31,32^. Overall, the modeled BC concentrations are in reasonable agreement with the measurements (correlation coefficient of 0.82, 8.2 ± 5.5 vs. 4.8 ± 3.9 μg m^−3^ in China, 1.4 ± 0.9 vs. 0.7 ± 0.7 μg m^−3^ in Korea, and 0.8 ± 0.9 vs. 0.4 ± 0.3 μg m^−3^ in Japan). There are relatively small differences in monthly averages of BC concentration between different years in Korea (0.8 ± 0.3 μg m^−3^ in 2001)^33^ and Japan (0.4 ± 0.4 μg m^−3^ from April 2009 to March 2015)^31^, while significant day-to-day variability is observed. These measurements suggest that the comparison of Fe concentrations between different years should be accompanied with day-to-day variability. Thus, we present a comparison of Fe in particulate matter with diameters below 2.5 μm (PM_2.5_) and total PM between modeled monthly averages with a standard deviation and measurements at the GOSAN site on Jeju Island in South Korea^33^, the East China Sea^34^ and the Bay of Bengal^35^ (Fig. 1). The range of one standard deviation is based on the daily averages of modeled Fe concentration in the same month as the measurements. The monthly mean values of our estimates of Fe concentration in total PM (i.e, PM_bin1–bin4_) tend to underestimate Fe concentration (0.40 ± 0.03 vs. 3.1 ± 3.4 μg m^−3^ in Korea, 0.2 ± 0.1 vs. 0.8 ± 1.0 μg m^−3^ in the East China Sea, and 0.2 ± 0.2 vs. 0.5 ± 0.4 μg m^−3^ in the Bay of Bengal), partly due to the episodic nature of dust events when compared to the model, which uses the year of 2014 for the assimilated meteorological data and emission source of Fe. Since Fe-containing aerosols from combustion sources are characterized by higher solubility in water and smaller size compared to mineral dust sources, Fe loading should be separately attributed to combustion and dust aerosols, due to their distinct emission sources and atmospheric processing. The modeled Fe concentration in PM_2.5_ indicates a fairly good agreement with the measurements of Fe (0.10 ± 0.08 vs. 0.10 ± 0.11 μg m^−3^ in the Bay of Bengal) especially at higher Fe solubilities.

**Figure 2:**
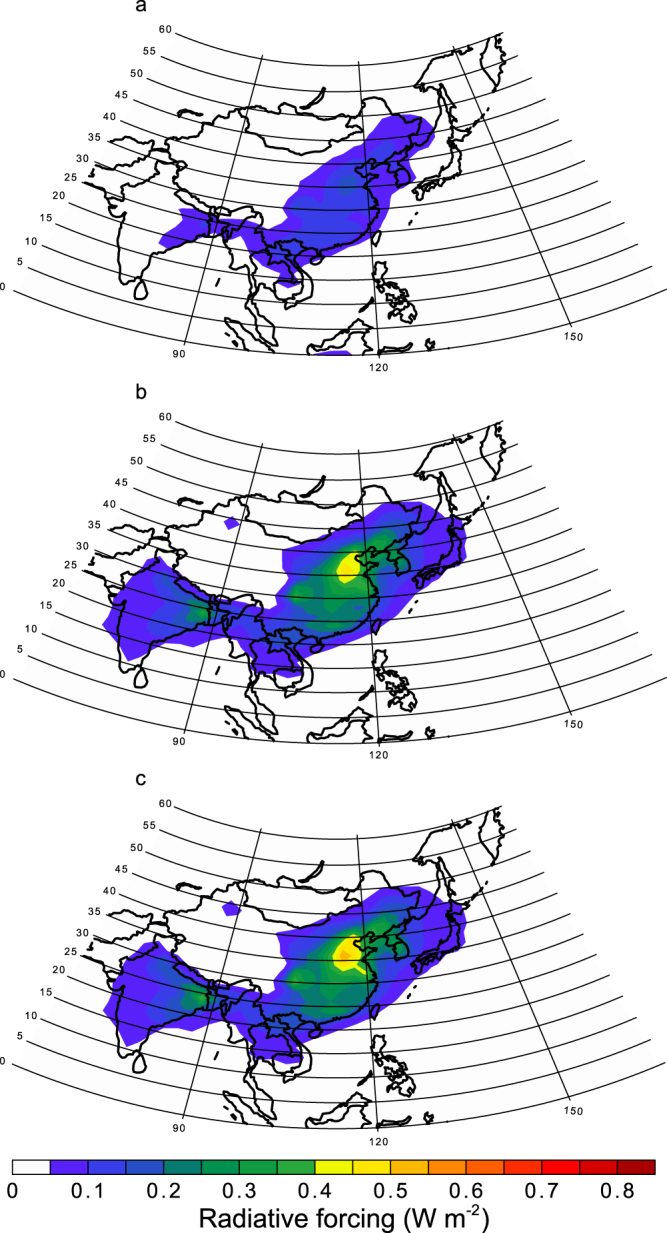
Annual mean of all-sky radiative forcing at the top of the atmosphere by light-absorbing aerosols of pyrogenetic Fe oxides. (**a**) Fe oxides are treated as magnetite in PM_bin1_. (**b**) Fe oxides are treated as magnetite in PM_bin2–bin4_ assuming internal mixing of pyrogenic Fe and other dust components in the RF calculation. (**c**) Fe oxides are treated as magnetite in PM_bin2–bin4_ assuming external mixing of pyrogenic Fe from other dust components in the RF calculation. This map was created in the Visual Climate Data Analysis Tools (VCDAT) version 4.1.2.

Aerosol RF is estimated as the difference in the calculated radiative fluxes with all aerosols and with all aerosols except the aerosol type being estimated in the RF calculation (Tables 1 and 2). Figure 2 shows the annual mean all-sky aerosol RF at the top of the atmosphere due to the absorption of strongly absorbing Fe oxides (magnetite) for particulate matter with diameters below (above) 1.26μm, PM_bin1_ (PM_bin2–bin4_). The RF for PM_bin2–bin4_ over polluted regions such as China exceeds 0.4 W m^−2^ (up to 0.5 W m^−2^), which is substantially larger than that for PM_bin1_ (less than 0.2 W m^−2^) regardless of the assumption in mixing state of pyrogenic Fe and other dust components in the RF calculation. Since the aerosol RF is estimated as the difference in the calculated radiative fluxes with all aerosols and with all aerosols except the aerosol type being estimated in the RF calculation, the effect of absorption by the other internally mixed compounds is subtracted even in case of volume-weighted homogeneous mixing of all aerosol species. Thus, the RF calculation of specific aerosols is not significantly affected by the assumed mixing state in the RF calculation.

**Table 1:** Summary of radiative transfer model experiments performed in this study.

Simulation	BC	Sub-micron Fe	Super-micron Fe	Super-micron Fe-containing minerals
Experiment 1	BC	Zero	Zero	Dust from soil
Experiment 2	Zero	Zero	Zero	Dust from soil
Experiment 3	BC	Magnetite	Zero	Dust from soil & combustion
Experiment 4	BC	Magnetite	Magnetite	Dust from soil & combustion
Experiment 5	BC	Hematite	Hematite	Dust from soil & combustion
Experiment 6	BC	Goethite	Goethite	Dust from soil & combustion
Experiment 7	BC	Zero	Zero	Dust from soil & combustion
Experiment 8	BC	Zero	Zero	Zero
Experiment 9	BC	Zero	Magnetite	Zero
Experiment 10	BC	Fe_3_O_4_ high	Fe_3_O_4_ high	Dust from soil & combustion
Experiment 11	BC	Fe_3_O_4_ low	Fe_3_O_4_ low	Dust from soil & combustion

Fe_3_O_4_ high (low) represents the use of high (low) refractive indices of magnetite (Fe_3_O_4_) examined by Zhang *et al*.^5^ (cited as Amaury *et al*. (unpublished data) and Querry^41^).

**Table 2:** Summary of radiative forcing calculated in this study.

Radiative effect	Difference
BC	Experiment 1–Experiment 2
Magnetite internal in TPM & super-micron combustion	Experiment 4–Experiment 1
Magnetite internal in PM_bin1_	Experiment 3–Experiment 7
Magnetite internal in TPM	Experiment 4–Experiment 7
Hematite	Experiment 5–Experiment 7
Goethite	Experiment 6–Experiment 7
Magnetite internal in super-micron combustion	Experiment 4–Experiment 3
Magnetite external in super-micron combustion	Experiment 9–Experiment 8
Fe_3_O_4_ high internal in TPM	Experiment 10–Experiment 7
Fe_3_O_4_ low internal in TPM	Experiment 11–Experiment 7

Internal (external) represents the use of internal (external) mixing of pyrogenic Fe and other dust components.

**Figure 3:**
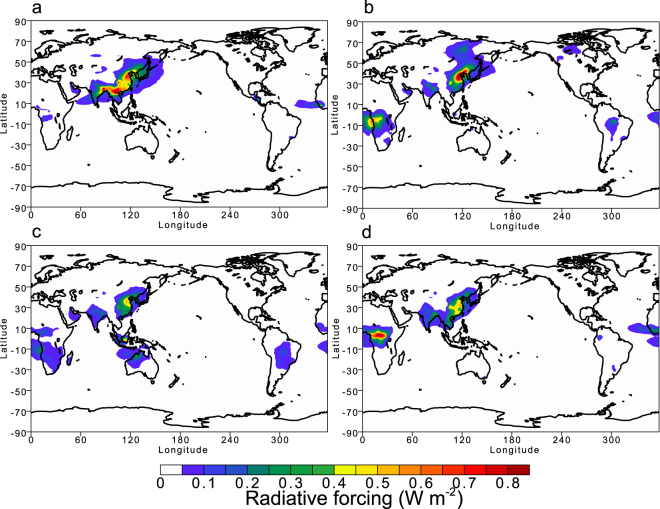
Seasonal averages of all-sky radiative forcing at the top of the atmosphere by light-absorbing aerosols of pyrogenetic Fe oxides (magnetite). (**a**) Spring (March, April, and May). (**b**) Summer (June, July, and August). (**c**) Autumn (September, October, and November). (**d**) Winter (December, January, and Feburary). This map was created in VCDAT version 4.1.2.

The seasonal averages of RF values for total particulate matter (TPM) over the polluted regions are comparable to those over the major biomass burning regions in central African counties and Indonesia where the Fe oxides (magnetite) in mineral dust might be entrained by pyro-convection (Fig. 3). Measurements for biomass burning events using the modified single-particle soot photometer (SP2)^21^ are needed to constrain the model estimates of pyrogenic Fe loading.

**Figure 4:**
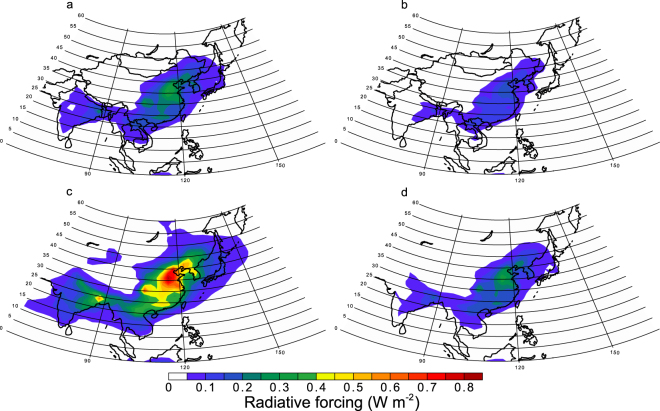
Annual mean of all-sky radiative forcing at the top of the atmosphere by light-absorbing aerosols of pyrogenetic Fe oxides. (**a**) Fe oxides are treated as hematite in TPM, which is considered as another form of Fe oxides at emission. (**b**) Fe oxides are treated as goethite in TPM, which is considered as a proxy of ferrihydrite. (**c**) Fe oxides are treated as magnetite in TPM using high refractive index. (**d**) Fe oxides are treated as magnetite in TPM using low refractive index. This map was created in VCDAT version 4.1.2.

Enhancements of RF over polluted regions by Fe-containing combustion aerosols depend on the treatment of Fe species in the aerosols (Fig. 4). The annual mean of RF for hematite is less than that for magnetite but still exceeds 0.2 (W m^−2^) over polluted regions in China, while that for goethite is less than 0.2 (W m^−2^). The Fe-rich phase of fly ash has been identified as magnetite and hematite, depending on whether the combustion conditions are reducing or oxidizing^8–11^. Thus hematite is considered as another form of Fe oxides at emission. On the other hand, the structure and associated absorption spectra of goethite and ferrihydrite (density of 3.8 g cm^−3^) are similar to each other^5,36^. Thus, for optical properties, goethite is considered as a proxy of ferrihydrite, which can be transformed from Fe-containing minerals during atmospheric processing. In urban aerosols, the major Fe oxides have been identified as hematite, goethite, and ferrihydrite by spectroscopic techniques^37,38^. However, the robustness and accuracy of the mass fraction of each Fe chemical species retrieved from the spectroscopic techniques has been called into question, because Fe speciation is not consistent with other studies possibly due to limitations in fitting the spectra of real aerosol samples with standard material spectra^39^. In order to estimate the effect of changes in Fe speciation with time for combustion aerosols, specific transformation rates of surface Fe nanoparticles and actual refractive indices for Fe-containing combustion aerosols are needed.

**Figure 5:**
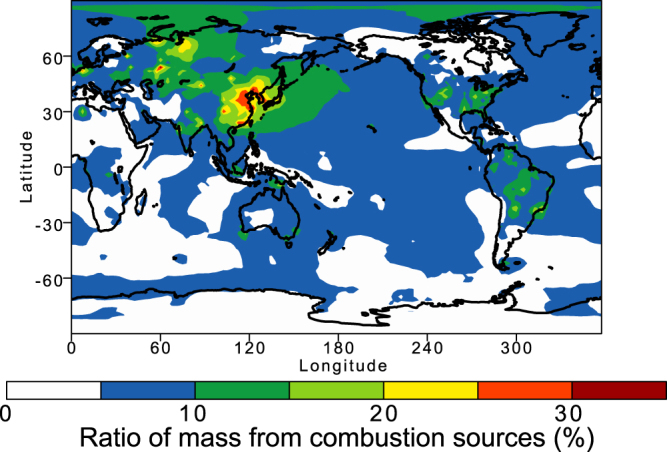
Annually averages of monthly averaged mass ratio of Fe/BC in aerosols in 2014. This map was created in VCDAT version 4.1.2.

Significant differences in the measurements of refractive indices between different studies may cause a major source of uncertainty in the RF calcualtion^5^. Therefore, in addition to the measurements of refractive index for magnetite^40^ used by Moteki *et al*.^21^, we used two data sets examined by Zhang *et al*.^5^ (cited as Amaury *et al*. (unpublished data) and Querry^41^). The resulting RF is slightly lower than that with the high refractive index of Amaury *et al*. (unpublished data) (Fig. 4c), but is significantly higher than that with lowest refractive index^41^ (Fig. 4d). Since the model-calculated aerosol mass ratio of Fe/BC was in reasonable agreement with the measurements using the modified SP2, which detected light-absorbing refractory aerosols of BC and Fe oxides, over the Yellow Sea and East China Sea^21^ (Fig. 5), the model-calculated light-absorbing Fe oxides are expected to be representative of the major Fe oxides from combustion sources in East Asia.

**Figure 6:**
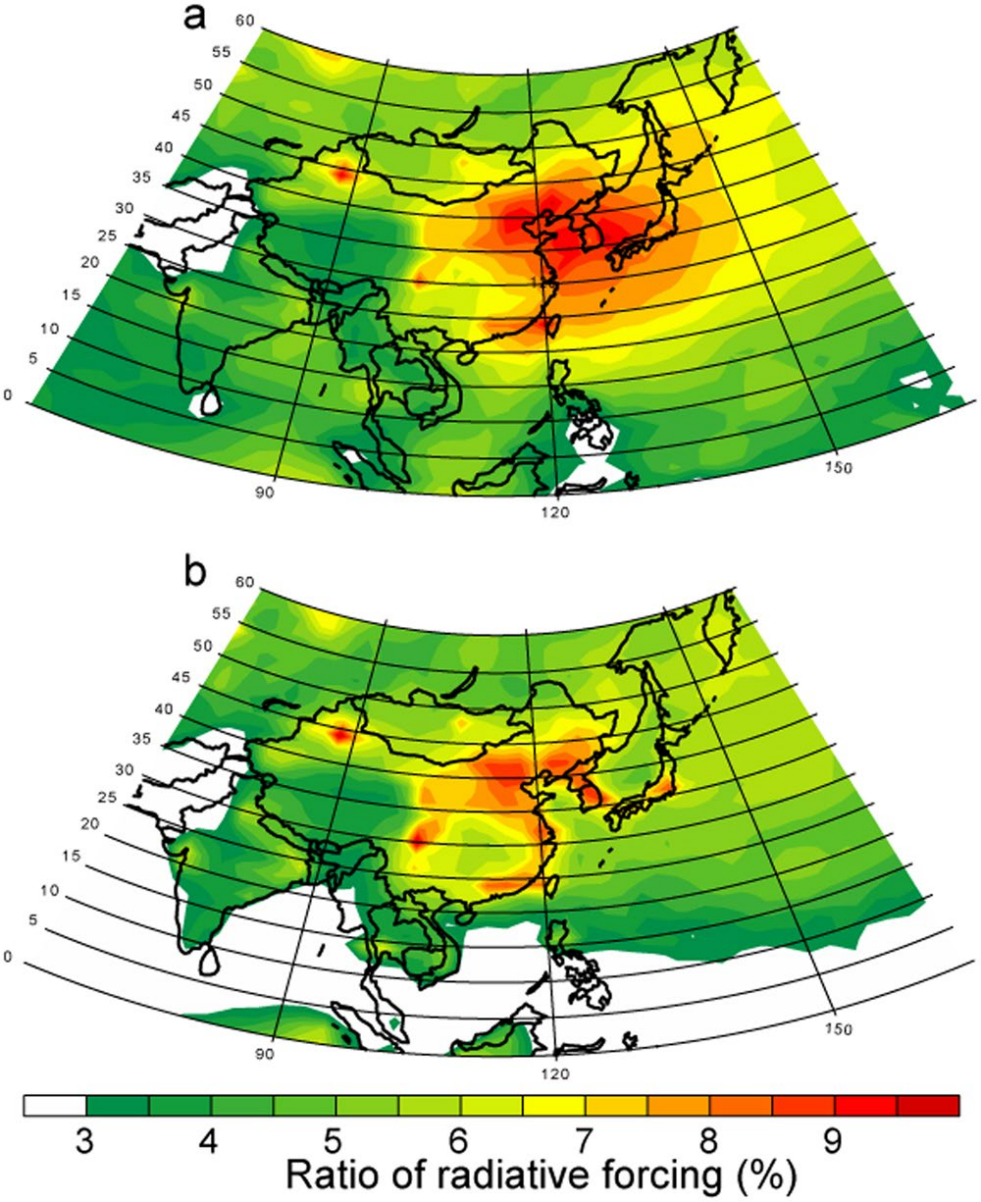
Ratio of annual mean of all-sky radiative forcing at the top of the atmosphere (%) in 2014. (**a**) (Magnetite)/BC. (**b**) (Magnetite + super-micron combustion dust)/BC. This map was created in VCDAT version 4.1.2.

The model results suggest that Fe oxides from combustion sources significantly contribute to a warming effect at the top of the atmosphere over air polluted regions in East Asia (from 5% to 10% of the RF due to BC), which is consistent with the shortwave absorption of magnetite relative to BC based on aircraft measurements^21^ (Fig. 6). In Urumqi, central Asia (44°N, 88°E), we found a significant contribution of the RF by Fe oxides to that of BC (about 10% except in winter for residential heating), due to high ratio of Fe/BC emissions for the energy (0.96) and industry (0.89) sectors (Table 3). The measurements of aerosols and surface snow samples demonstrated that Fe-dominant spherical particles were mainly in super-micron aerosols and originated from iron and steel plants^42,43^. The Fe content of aerosols from the iron and steel industries (26%) is significantly higher than that from coal fly ash (6.4%)^44,45^ (Table 4).

**Table 3:** Comparison of anthropogenic emissions between Fe and BC (Tg yr^−1^) for each sector.

Sector	Fe	BC^55^	Fe/BC
Energy	1.1(59%)	1.2(16%)	0.96
Industrial	0.7(39%)	0.8(11%)	0.89
Residential, commercial, others	0.0(0%)	3.7(50%)	0.00
Waste	0.0(1%)	0.1(1%)	0.31
Transport including shipping	0.0(1%)	1.6(21%)	0.01
Total	1.9	7.4	0.26

Values in the parentheses represent the percentage of each sector to total. Fe includes sub-micron and super-micron particles.

**Table 4:** Summary of parameters used to estimate Fe emissions in this study.

Sector	Fuel	$\frac{{{\boldsymbol{F}}}_{{\boldsymbol{P}}{\boldsymbol{I}}{\boldsymbol{M}}}}{{{\boldsymbol{F}}}_{{\boldsymbol{B}}{\boldsymbol{C}}}{\boldsymbol{+}}{{\boldsymbol{F}}}_{{\boldsymbol{P}}{\boldsymbol{O}}{\boldsymbol{M}}}}$	*F*_1_	*F*_*sub*–*micron Fe*_	*F*_*super*–*micron Fe*_
Energy	FF	0.30	0.19	6.4%	6.4%
Industrial	FF	0.30	0.19	6.4%	6.4%
Iron and Steel	FF + BF	0.30	0.19	26%	26%
Residential	FF	0.037	0.90	0.10%	0.10%
Waste	FF	0.31	0.82	0.36%	1.02%
Shipping	FF	3.5	0.86	0.96%	1.7%

FF stands for fossil fuel combustion. BF represents biofuel combustion. Fractions of BC (*F*_*BC*_), POM (*F*_*POM*_), the emissions with diameters below 1 μm (*F*_1_), and Fe content for sub-micron (*F*_*sub-micron Fe*_) and super-micron (*F*_*super-micron Fe*_) particles are taken from compilation of measurements^28,29,44,45,56–58^. Fraction of particulate inorganic matter in sub-micron size (*F*_*PIM*_) is calculated using equation (2).

**Figure 7:**
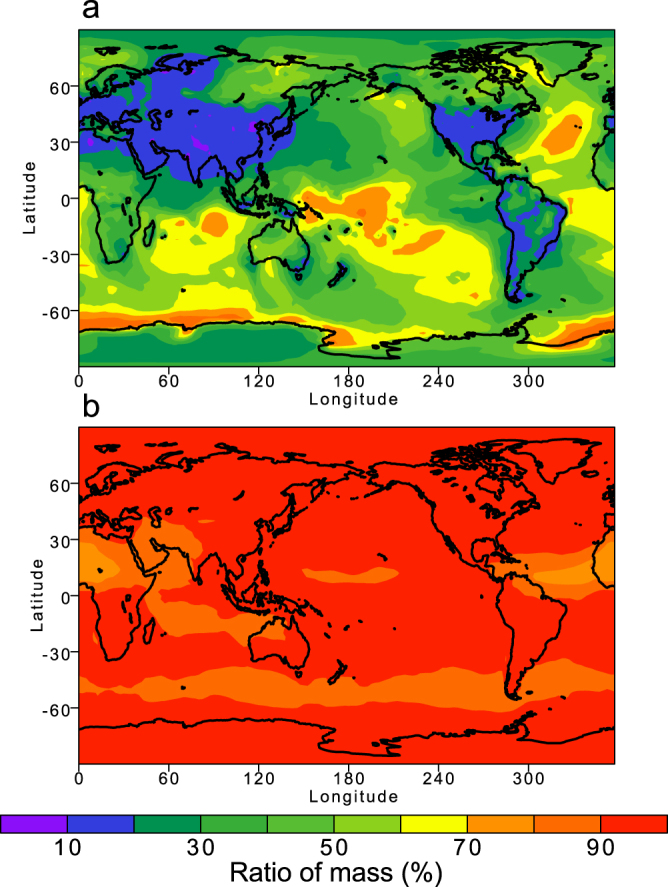
Annual mean of mass fraction (%) in PM_bin1_ relative to that in TPM in 2014. (**a**) Pyrogenic Fe. (**b**) Sulfate. This map was created in VCDAT version 4.1.2.

In addition to the surface enrichment of Fe aggregates, Fe oxides are internally mixed with amorphous aluminosilicate^8–11^ and the Fe-containing aerosols can be coated by sulfate. Thus, the effect of Fe-containing aerosols may depend on treatment of mixing with other types of super-micron combustion aerosols (i.e., amorphous aluminosilicate) which are co-emitted with precursor gases and then can be coated by secondary formation materials (e.g., sulfate) in the atmosphere (Fig. 6b). The relative contribution of RF due to Fe oxides is smaller than 6% because of less light-absorbing aluminosilicate minerals over the ocean but is still significant over polluted regions (about 10% of the RF due to BC). Compared to Fe aggregates, the relative contribution of RF due to Fe oxides is not significantly enhanced over the land, partly because most sulfates (pyrogenic Fe oxides) reside in the sub-micron (super-micron) particles (Fig. 7) even though the internal mixing of light-absorbing aerosols with sulfate can enhance the absorption of solar radiation^46^. Thus our model results support the hypothesis that light absorption due to aerosols is enhanced partially due to Fe oxides from pyrogenetic Fe sources in air polluted regions.

## Implications

The light-absorbing Fe oxides may heat the atmosphere in polluted regions, while atmospheric processing of Fe oxides can reduce its heating capacity by transforming strongly light-absorbing Fe oxides (i.e., magnetite and hematite) to weakly absorbing species (i.e., ferrihydrite) during long-range transport. These nanoparticles are also important as bioavailable Fe which affects both human and ocean health. Thus, reducing Fe-containing aerosol emissions by proper equipment of the emission control device will benefit both climate and our health. As such, our results highlight the need for improving the process-based understanding of the effects of emission sources and chemical transformation of pyrogenetic Fe oxides on both optical properties and bioavailability. Since a rapid growth in energy consumption by the iron and steel industries in newly developing countries is projected in the next decades^47^, this is especially crucial for assessing the future impact of air quality changes on climate and ecosystems.

## Methods

This study uses the Integrated Massively Parallel Atmospheric Chemical Transport (IMPACT)^48^ model to calculate the concentration of aerosols^49^ and reactive gaseous species^50^. The model is driven by the Goddard Earth Observation System–Forward Processing (GEOS-FP) assimilated meteorological data from of the NASA Global Modeling and Assimilation Office (GMAO)^51^ with a horizontal resolution of 2.0° × 2.5° and 59 vertical layers for the year of 2014. The three-dimensional model simulates the emissions, chemistry, transport, and deposition of major aerosol species, which includes BC, particulate organic matter (POM), mineral dust, sulfate, nitrate, ammonium, and sea spray aerosols, and their precursor gases. We calculated dust emissions using a physically-based emission scheme (Experiments 3 in Ito and Kok^52^). A mineralogical map^53^ was used to estimate the emissions of Fe in aeolian dust^54^. Atmospheric processing of Fe-containing aerosols are predicted in 4 size bins (diameters: <1.26, 0.126–2.5, 2.5–5, and 5–20 µm)^14^. The transport and deposition of the sub-micron (super-micron) Fe particles from combustion sources are treated similarly to BC (super-micron dust)^28,44^. Transformation from insoluble Fe to labile Fe in aerosol water due to proton-promoted, oxalate-promoted, and photo-reductive Fe dissolution schemes is dynamically simulated for the size-segregated mineral dust^54^ and combustion aerosols^14^. Atmospheric concentrations of total and labile Fe in aerosols have been evaluated extensively on global and regional scales^14,29,54^.

We updated the emission data from anthropogenic sources, following the emission data set distributed for the Intergovernmental Panel on Climate Change (IPCC) report. The monthly emission data sets for anthropogenic activities such as fossil fuel use and biofuel combustion are taken from the Community Emission Data System (CEDS)^55^. The sub-micron particulate inorganic matter (PIM) emissions (*E* (c, s, f, t)_*PIM*_) from anthropogenic combustion sources are estimated using BC and organic carbon (OC) emissions and fraction of particulate inorganic matter (*F* (s, f)_*PIM*_) in sub-micron size, according to following:

1 $$E{({\rm{c}},{\rm{s}},{\rm{f}},{\rm{t}})}_{PIM}=(E{({\rm{c}},{\rm{s}},{\rm{f}},{\rm{t}})}_{BC}+E{({\rm{c}},{\rm{s}},{\rm{f}},{\rm{t}})}_{POM})\times \frac{F{({\rm{s}},{\rm{f}})}_{PIM}}{F{({\rm{s}},{\rm{f}})}_{BC}+F{({\rm{s}},{\rm{f}})}_{POM}}$$

2 $$F{({\rm{s}},{\rm{f}})}_{PIM}=1-(F{({\rm{s}},{\rm{f}})}_{BC}+F{({\rm{s}},{\rm{f}})}_{POM})$$

where POM = 1.3 × OC, c is country, s is sector, f is fuel (where applicable), and t is time. Since the Fe content of aerosols from the iron and steel industries (26%) is higher than that from coal fly ash (6.4%), this sector is treated separately^44,45^. The emissions of total particulate matter (TPM) are calculated by dividing those of PM_1_ by *F* (s, f)_1_, which is the fraction of the emissions with diameters below 1 μm^29,56–58^. The sub-micron (super-micron) Fe emissions are estimated by multiplying PM_1_ (TPM–PM_1_) by *F* (s, f)_*sub*–*micron Fe*_ (*F* (s, f)_*super*–*micron Fe*_), which is the Fe content for sub-micron (super-micron) particles. Table 4 summarizes the parameters used to estimate anthropogenic emissions of Fe from combustion sources in this study.

We estimate daily emissions of particulate matter and their precursor gases from open biomass burning which are derived from satellite measurements and a biogeochemical model^59,60^. We upgraded the spatially explicit individual-based dynamic global vegetation model (SEIB-DGVM) to estimate the carbon density over vegetated lands^61,62^. We also updated the satellite measurements of the MODIS burned areas (MCD64A1)^63^, fractional vegetation cover (MOD44B)^64^, vegetation indices (MOD13A1)^65^, and land cover data set (MCD12Q1)^66^. Our estimates of fuel consumption showed reasonable agreement with the compilation of field measurements^67^ (Table 5). Over all, our estimates of Fe emissions were consistent with previous estimates (Table 6)^28–30^. Fe oxides emitted from combustion sources largely reside in super-micron aerosols, which are also consistent with the measurements^21^ (Fig. 7).

**Table 5:** Comparison of fuel consumption (kg m^−2^) between model estimates in this study and compilation of field measurements^67^, and their differences (%).

Biome	This study	Measurements^62^	Difference (%)
Tropical forest	12.0	12.6	−5%
Temperate forest	5.4	5.8	−7%
Boreal forest	3.9	3.5	11%
Shrub	3.7	2.8	32%
Woodland	0.65	0.46	41%
Grassland	0.40	0.43	−7%
Crop	0.34	0.65	−48%
Tropical peat	38.0	31.4	21%
Boreal peat	1.5	4.2	−64%
Wetland	0.47

**Table 6:** Comparison of Fe emissions from combustion sources (Tg Fe yr^−1^) in different studies.

Study	Fossil fuel and biofuel combustion		Biomass burning	
	Sub-micron	Super-micron	Sub-micron	Super-micron
This work	0.11	1.8	0.13	0.66
Luo *et al*.^28^	0.10	0.56	0.21	0.86
Ito^29^	0.07	0.44	0.23	0.92
Wang *et al*.^30^	0.038	1.0 (3.2)	0.017	0.46 (0.38)

The values in parentheses show Fe emissions in PM >10 μm.

We use an off-line radiative transfer model to calculate the optical properties of aerosols and their resulting RF, based on the Lawrence Livermore National Laboratory (LLNL) Solar Radiative Transfer Model (SRTM)^68^. In this off-line radiative transfer model, the aerosols can be treated as either internally mixed or externally mixed in each size bin^69^. We used daily averaged aerosol concentration together with four-hourly meteorological fields to estimate instantaneously varying optical properties, which vary with water uptake by the aerosols. Four-hourly aerosol optical properties are calculated using a look-up table as a function of wavelength and size parameter. Five types of aerosols (i.e., carbonaceous aerosols from anthropogenic combustion, carbonaceous aerosols from open biomass burning, dust, sulfate, and sea salt) were assumed to be externally mixed in each size bin, while sulfate coated on each aerosol was internally mixed within each aerosol type and size bin^69^. The refractive index for internally mixed aerosols is calculated based on the volume weighted mixture for each aerosol type and size bin.

Here, we calculated the volume weighted averages of refractive indices as mixtures of the sub-micron Fe oxides and carbonaceous aerosols from anthropogenic combustion and open biomass burning, respectively, while those of the super-micron Fe-containing minerals and dust for the 3 super-micron size bins, were estimated separately in the radiative transfer model. Although common simplification to treat super-micron aerosols as spherical particles may contribute to additional uncertainty in the RF calculation for aspherical dust particles, the fly ashes from power plants^10,11^, biomass burning^24^, and iron and steel plants^42,43^ are often observed as spherical particles. The size-segregated Fe-containing aerosols can also be coated by sulfate as a result of condensation of sulfate gas, coagulation of each particle with sulfate particles, and through the formation of sulfate in cloud water which forms on different cloud condensational nuclei (CCN) particles^14,49^. We treat Fe oxides as magnetite, hematite, and goethite separately to estimate the RF of each Fe oxide (Tables 1 and 2). Here, we use the same refractive indices for BC^70^ and magnetite^40^ as those used by Moteki *et al*.^21^. In sensitivity simulations, we used two data sets examined by Zhang *et al*.^5^ (cited as Amaury *et al*. (unpublished data) and Querry^41^). The refractive index for hematite is the same as that used by Scanza *et al*.^3^ (cited as personal communication with A. H. M. J. Triaud, 2005). The refractive index for goethite is taken from Bedidi and Cervelle^6^. The bulk densities of magnetite (5.17 g cm^−3^), hematite (5.3 g cm^−3^), and goethite (3.8 g cm^−3^) are used in the radiative transfer model.

## Code and data availability

The off-line radiative transfer model and the output data generated during the current study are available from the corresponding author on reasonable request.

## References

[1] Tegen I (1997). Contribution of different aerosol species to the global aerosol extinction optical thickness: Estimates from model results. J. Geophys. Res..

[2] Balkanski Y, Schulz M, Claquin T, Guibert S (2007). Reevaluation of Mineral aerosol radiative forcings suggests a better agreement with satellite and AERONET data. Atmos. Chem. Phys..

[3] Scanza RA (2015). Modeling dust as component minerals in the Community Atmosphere Model: development of framework and impact on radiative forcing. Atmos. Chem. Phys..

[4] Sokolik IN, Toon OB (1999). Incorporation of mineralogical composition into models of the radiative properties of mineral aerosol from UV to IR wavelengths. J. Geophys. Res..

[5] Zhang XL, Wu GJ, Zhang CL, Xu TL, Zhou QQ (2015). What is the real role of iron oxides in the optical properties of dust aerosols?. Atmos. Chem. Phys..

[6] Bedidi A, Cervelle B (1993). Light scattering by spherical particles with hematite and goethitelike optical properties: Effect of water impregnation. J. Geophys. Res..

[7] Lafon S, Sokolik IN, Rajot JL, Caquineau S, Gaudichet A (2006). Characterization of iron oxides in mineral dust aerosols: Implications for light absorption. J. Geophys. Res..

[8] Hansen LD, Silberman D, Fisher GL (1981). Crystalline components of stack-collected, size-fractionated coal fly ash. Environ. Sci. Technol..

[9] Hansen LD, Silberman D, Fisher GL, Eatough DJ (1984). Chemical speciation of elements in stack-collected, respirable-size, coal fly ash. Environ. Sci. Technol..

[10] Norton GA, Markuszewski R, Shanks HR (1986). Morphological and chemical characterization of iron-rich fly ash fraction. Environ. Sci. Technol..

[11] Blaha U, Sapkota B, Appel E, Stanjek H, Rosler W (2008). Micro-scale grain-size analysis and magnetic properties of coal-fired power plant fly ash and its relevance for environmental magnetic pollution studies. Atmos. Environ..

[12] Veranth JM (2000). Mössbauer spectroscopy indicates that iron in an aluminosilicate glass phase is the source of the bioavailable iron from coal fly ash. Chem. Res. Toxicol..

[13] Chen HH, Grassian VH (2013). Iron dissolution of dust source materials during simulated acidic processing: The effect of sulfuric, acetic, and oxalic acids. Environ. Sci. Technol..

[14] Ito A (2015). Atmospheric processing of combustion aerosols as a source of bioavailable iron. Environ. Sci. Technol. Lett..

[15] Maher, J. M. *et al*. Magnetite pollution particles in the human brain. *P. Natl. Acad. Sci*. **114**, 10797–10801. 10.1073/pnas.1605941113 PMC5047173 27601646

[16] Schroth AW, Crusius J, Sholkovitz ER, Bostick BC (2009). Iron solubility driven by speciation in dust sources to the ocean. Nat. Geosci..

[17] Fu H (2012). Solubility of iron from combustion source particles in acidic media linked to iron speciation. Environ. Sci. Technol..

[18] Fang T (2017). Highly acidic ambient particles, soluble metals, and oxidative potential: a link between sulfate and aerosol toxicity. Environ. Sci. Technol..

[19] Li WJ (2017). Air pollution-aerosol interactions produce more bioavailable iron for ocean ecosystems. Sci. Adv..

[20] Shi ZB (2009). Formation of iron nanoparticles and increase in iron reactivity in the mineral dust during simulated cloud processing. Environ. Sci. Technol..

[21] Moteki N (2017). Anthropogenic iron oxide aerosols enhance atmospheric heating. Nat. Commun..

[22] Oldfield F, Thompson R, Dickson DPE (1981). Artificial magnetic enhancement of stream bedload: a hydrological application of superparamagnetism. Phys. Earth Planet. Inter..

[23] Clement BM, Javier J, Sah JP, Ross MS (2011). The effects of wildfires on the magnetic properties of soils in the Everglades. Earth Surf. Process. Landforms.

[24] Takahama S, Gilardoni S, Russell LM (2008). Single-particle oxidation state and morphology of atmospheric iron aerosols. J. Geophys. Res..

[25] Certini G (2005). Effects of fire on properties of forest soils: a review. Oecologia.

[26] Hanesch M, Stanjek H, Petersen N (2006). Thermomagnetic measurements of soil iron minerals: the role of organic carbon. Geophys. J. Int..

[27] Till JL, Guyodo Y, Lagroix F, Morin G, Ona-Nguema G (2015). Goethite as a potential source of magnetic nanoparticles in sediments. Geology.

[28] Luo C (2008). Combustion iron distribution and deposition. Global Biogeochem. Cycles.

[29] Ito A (2013). Global modeling study of potentially bioavailable iron input from shipboard aerosol sources to the ocean. Global Biogeochem. Cycles.

[30] Wang R (2015). Sources, transport and deposition of iron in the global atmosphere. Atmos. Chem. Phys..

[31] Kanaya Y (2016). Long-term observations of black carbon mass concentrations at Fukue Island, western Japan, during 2009–2015: constraining wet removal rates and emission strengths from East Asia. Atmos. Chem. Phys..

[32] Fang, W. *et al*. Divergent evolution of carbonaceous aerosols during dispersal of East Asian haze. *Sci. Rep*. **7** (2017). 10.1038/s41598-017-10766-4 PMC5585391 28874801

[33] Duvall RM (2008). The water-soluble fraction of carbon, sulfur, and crustal elements in Asian aerosols and Asian soils. Atmos. Environ..

[34] Hsu S-C (2010). Sources, solubility, and dry deposition of aerosol trace elements over the East China Sea. Mar. Chem..

[35] Kumar A, Sarin MM, Srinivas B (2010). Aerosol iron solubility over Bay of Bengal: Role of anthropogenic sources and chemical processing. Mar. Chem..

[36] Glotch TD, Rossman GR (2009). Mid-infrared reflectance spectra and optical constants of six iron oxide/oxyhydroxide phases. Icarus.

[37] Oakes M (2012). Iron solubility related to particle sulphur content in source emission and ambient fine particles. Environ. Sci. Technol..

[38] Kurisu M, Takahashi Y, Iizuka T, Uematsu M (2016). Very low isotope ratio of iron in fine aerosols related to its contribution to the surface ocean. J. Geophys. Res..

[39] Shi ZB (2012). Impacts on iron solubility in the mineral dust by processes in the source region and the atmosphere: A review. Aeolian Res..

[40] Huffman, D. R. & Stapp, J. L. Optical Measurements on Solids of Possible Interstellar *Importance in Interstellar Dust and Related Topics*, J. M. Greenberg and H. C. Van de Hulst, eds Reidel, Boston, pp. 297–301 (1973).

[41] Querry, M. R. Optical Constants, Contractor report, US Army Chemical Research, Development and Engineering Center (CRDC), Aberdeen Proving Ground, MD, 418 pp (1985).

[42] Zhang XL, Wu GJ, Yao TD, Zhang CL, Yue YH (2011). Characterization of individual fly ash particles in surface snow at Urumqi Glacier No. 1, Eastern Tianshan. Chin. Sci. Bull..

[43] Yin H, Mu SY, Zhao L, Qi XL, Pan XL (2013). Microscopic morphology and elemental composition of size distributed atmospheric particulate matter in Urumqi, China. Environ. Earth Sci..

[44] Ito A, Feng Y (2010). Role of dust alkalinity in acid mobilization of iron. Atmos. Chem. Phys..

[45] Lin YC, Chen JP, Ho TY, Tsai IC (2015). Atmospheric iron deposition in the northwestern Pacific Ocean and its adjacent marginal seas: The importance of coal burning. Global Biogeochem. Cycles.

[46] Fuller KA, Malm WC, Kreidenweis SM (1999). Effects of mixing on extinction by carbonaceous particles. J. Geophys. Res..

[47] van Ruijven BJ (2016). Long-term model-based projections of energy use and CO2 emissions from the global steel and cement industries. Resources Conservation and Recycling.

[48] Rotman DA (2004). IMPACT, the LLNL 3-D global atmospheric chemical transport model for the combined troposphere and stratosphere: Model description and analysis of ozone and other trace gases. J. Geophys. Res..

[49] Liu XH, Penner JE, Herzog M (2005). Global modeling of aerosol dynamics: Model description, evaluation, and interactions between sulfate and nonsulfate aerosols. J. Geophys. Res..

[50] Ito A, Sillman S, Penner JE (2007). Effects of additional nonmethane volatile organic compounds, organic nitrates, and direct emissions of oxygenated organic species on global tropospheric chemistry. J. Geophys. Res..

[51] Lucchesi, R. File Specification for GEOS-5 FP, GMAO Office Note No. 4 (Version 1.1), available at https://gmao.gsfc.nasa.gov/GMAO_products/ (2017).

[52] Ito A, Kok JF (2017). Do dust emissions from sparsely vegetated regions dominate atmospheric iron supply to the Southern Ocean?. J. Geophys. Res..

[53] Journet E, Balkanski Y, Harrison SP (2014). A new data set of soil mineralogy for dust-cycle modeling. Atmos. Chem. Phys..

[54] Ito A, Shi Z (2016). Delivery of anthropogenic bioavailable iron from mineral dust and combustion aerosols to the ocean. Atmos. Chem. Phys..

[55] Hoesly RM (2018). Historical (1750–2014) anthropogenic emissions of reactive gases and aerosols from the Community Emission Data System (CEDS). Geosci. Model Dev..

[56] Bond TC (2004). A technology-based global inventory of black and organic carbon emissions from combustion. J. Geophys. Res..

[57] Bond TC (2007). Historical emissions of black and organic carbon aerosol from energy-related combustion, 1850–2000. Global Biogeochem. Cycles.

[58] Ito A, Penner JE (2005). Historical emissions of carbonaceous aerosols from biomass and fossil fuel burning for the period 1870–2000. Global Biogeochem. Cycles.

[59] Ito A, Penner JE (2004). Global estimates of biomass burning emissions based on satellite imagery for the year 2000. J. Geophys. Res..

[60] Ito A (2011). Mega fire emissions in Siberia: potential supply of bioavailable iron from forests to the ocean. Biogeosciences.

[61] Sato H, Itoh A, Kohyama T (2007). SEIB-DGVM: A new dynamic global vegetation model using a spatially explicit individual-based approach. Ecol. Model..

[62] Sato H, Kumagai T, Takahashi A, Katul GG (2015). Effects of different representations of stomatal conductance response to humidity across the African continent under warmer CO2-enriched climate conditions. J. Geophys. Res..

[63] Giglio L, Randerson JT, van der Werf GR (2013). Analysis of daily, monthly, and annual burned area using the fourth-generation global fire emissions database (GFED4). J. Geophys. Res..

[64] Hansen MC (2003). Global percent tree cover at a spatial resolution of 500 meters: First results of the MODIS vegetation continuous fields algorithm. Earth Interact..

[65] Huete A (2002). Overview of the radiometric and biophysical performance of the MODIS vegetation indices. Rem. Sens. Environ..

[66] Friedl MA (2010). MODIS Collection 5 global land cover: Algorithm refinements and characterization of new datasets. Rem. Sens. Environ..

[67] van Leeuwen TT (2014). Biomass burning fuel consumption rates: a field measurement database. Biogeosciences.

[68] Grant KE, Chuang CC, Grossman AS, Penner JE (1999). Modeling the spectral optical properties of ammonium sulfate and biomass burning aerosols: Parameterization of relative humidity effects and model results. Atmos. Environ..

[69] Xu L, Penner JE (2012). Global simulations of nitrate and ammonium aerosols and their radiative effects. Atmos. Chem. Phys..

[70] Bergstrom RW (1972). Predictions of the spectral absorption and extinction coefficients of an urban air pollution aerosol model. Atmos. Environ..

